# Optimized Blanching Reduces the Host Cell Protein Content and Substantially Enhances the Recovery and Stability of Two Plant-Derived Malaria Vaccine Candidates

**DOI:** 10.3389/fpls.2016.00159

**Published:** 2016-02-17

**Authors:** Stephan Menzel, Tanja Holland, Alexander Boes, Holger Spiegel, Johanna Bolzenius, Rainer Fischer, Johannes F. Buyel

**Affiliations:** ^1^Integrated Production Platforms, Fraunhofer-Institute for Molecular Biology and Applied Ecology IMEAachen, Germany; ^2^Plant Biotechnology, Fraunhofer-Institute for Molecular Biology and Applied Ecology IMEAachen, Germany; ^3^Institute for Molecular Biotechnology, RWTH Aachen UniversityAachen, Germany

**Keywords:** blanching, design of experiments, downstream processing, heat precipitation, host cell proteins, plant-derived biopharmaceuticals, vaccine development

## Abstract

Plants provide an advantageous expression platform for biopharmaceutical proteins because of their low pathogen burden and potential for inexpensive, large-scale production. However, the purification of target proteins can be challenging due to issues with extraction, the removal of host cell proteins (HCPs), and low expression levels. The heat treatment of crude extracts can reduce the quantity of HCPs by precipitation thus increasing the purity of the target protein and streamlining downstream purification. In the overall context of downstream process (DSP) development for plant-derived malaria vaccine candidates, we applied a design-of-experiments approach to enhance HCP precipitation from *Nicotiana benthamiana* extracts generated after transient expression, using temperatures in the 20–80°C range, pH values of 3.0–8.0 and incubation times of 0–60 min. We also investigated the recovery of two protein-based malaria vaccine candidates under these conditions and determined their stability in the heat-treated extract while it was maintained at room temperature for 24 h. The heat precipitation of HCPs was also carried out by blanching intact plants in water or buffer prior to extraction in a blender. Our data show that all the heat precipitation methods reduced the amount of HCP in the crude plant extracts by more than 80%, simplifying the subsequent DSP steps. Furthermore, when the heat treatment was performed at 80°C rather than 65°C, both malaria vaccine candidates were more stable after extraction and the recovery of both proteins increased by more than 30%.

## Introduction

Plants have been used to produce biopharmaceutical proteins for more than 25 years (Hiatt et al., 1989). Even though plant-based expression platforms offer inexpensive USP, scalability and a low human pathogen load (Commandeur et al., 2003; Schillberg et al., 2005; Fischer et al., 2012), only two products had received regulatory approval by 2015 and only one is currently on the market (Yusibov and Rabindran, 2008; Pastores et al., 2014). One reason for the delayed transition from concept to market is the erstwhile lack of regulatory guidance, which has been addressed by the authorities only in the last few years (CPMP, 2002; FDA, 2002; Fischer et al., 2012; Buyel et al., 2015b). The recombinant protein expression levels that can be achieved in plants were initially low, but have now reached the gram product per kilogram biomass range (Bendandi et al., 2010; Buyel, 2015). Combined with these improvements in USP, the often challenging purification of target proteins from plant extracts (Buyel and Fischer, 2014d) means that DSP can account for up to 80% of the total production costs (Wilken and Nikolov, 2012). This issue is especially pressing if no affinity purification step is available. Therefore, several genetic fusion tags have been developed to facilitate the purification of target proteins, including elastin-like polypeptides (Phan et al., 2013), hydrophobins (Reuter et al., 2014), and oleosin (Markley et al., 2006). However, the fusion tags can affect the functionality of the product and the fusion proteins attract regulatory scrutiny (Fischer et al., 2012). Process-based purification approaches solely exploiting the different intrinsic properties of the products and HCPs are therefore more appealing and should offer a higher success rate.

**FIGURE 1:**
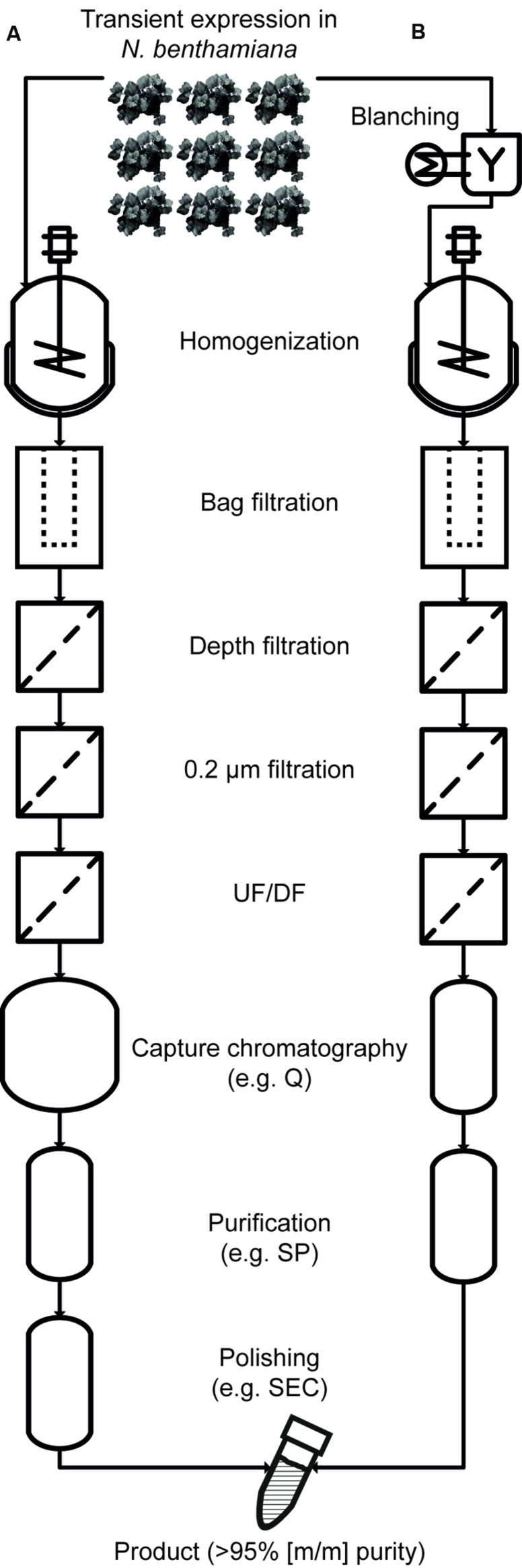
**Comparison of purification processes without heat treatment (A) and with blanching (B).** With the exception of the initial blanching steps, the process schemes are largely identical during extraction, clarification and concentration, but the size of the capture column can be reduced to reflect the depletion of HCPs. Also, the higher starting purity after extraction allows the number of chromatography-based purification steps to be reduced. UF/DF, ultrafiltration/diafiltration; Q, quaternary ammonium ligand (an anion exchange resin); SP, sulfopropyl (a cation exchange ligand); SEC, size exclusion chromatography.

Methods for selective product extraction (Turpen, 1999), secretion (Drake et al., 2009), and precipitation (Holler et al., 2007) have been developed along with strategies to remove HCPs, e.g., by low-pH extraction (Azzoni et al., 2002; Hassan et al., 2008; Buyel and Fischer, 2014d). Recently, the heat treatment of intact leaves in water baths (blanching) and the heating of plant extracts (Buyel and Fischer, 2014e; Buyel et al., 2014; Voepel et al., 2014; Beiss et al., 2015) have been used to selectively precipitate tobacco HCPs, increasing the initial purity of vaccine candidate proteins several fold. This facilitates subsequent recovery (Winkelnkemper and Schembecker, 2010) and reduces DSP costs. Specifically, there are no ‘platform’ ligands available for the purification of vaccine candidate proteins by affinity chromatography, analogous to the protein A ligands used to capture antibodies. Therefore, a combination of conventional chromatography steps, e.g., ion exchange chromatography, must be used for purification, typically aiming to exceed a final purity of 95% (m/m; Betancourt et al., 2007; Okuno et al., 2011; Buyel et al., 2012). This level of purity can be facilitated by the heat precipitation of otherwise highly abundant HCPs that can interfere with chromatography (Buyel and Fischer, 2014d). This approach also reduces manufacturing costs, which are especially important for products that will be predominantly used in developing countries, e.g., malaria vaccines. An overview of a DSP process using blanching is shown in **Figure 1**.

Together with tuberculosis and HIV, the poverty-related infectious disease malaria (caused by the parasite *Plasmodium falciparum*) remains a major burden for developing countries, accounting for 660 000–1 200 000 deaths worldwide, mostly affecting children in sub-Saharan Africa (Murray et al., 2012). Even though the first malaria vaccine, a PfCSP-based vaccine addressing the pre-erythrocytic stage of the parasite (Mosquirix, developed by GSK), has completed phase III clinical trials (Wilby et al., 2012), it has not been recommended for widespread deployment by the World Health Organization (Callaway and Maxmen, 2015). Thus, we still not only lack a vaccine but also a formulation that can induce long-lasting protection in all vaccinated individuals.

**FIGURE 2:**
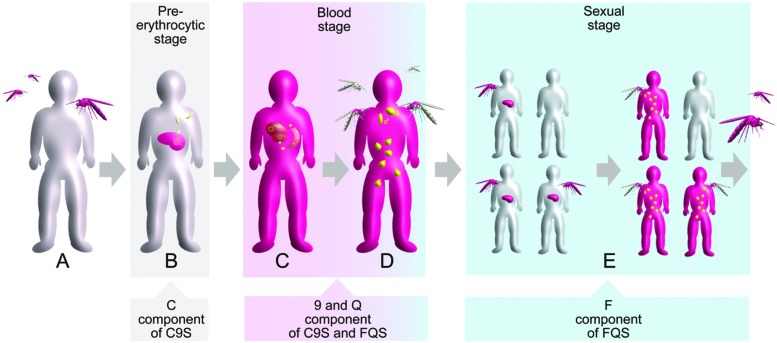
**Schematic overview of the *Plasmodium falciparum* life cycle in the context of human infection and transmission.** The three main stages are indicated by colored boxes. Gray indicates healthy status whereas pink indicates infected status is. Different forms of the parasite are shown in yellow. Target points of the vaccine components are indicated by arrowed boxes. **(A)** Healthy individual. **(B)** Infection of liver cells after a mosquito bite. **(C)** Manifestation of blood-stage parasitemia. **(D)** Formation of gametocytes initiating the sexual stage. **(E)** Parasite transmission within a human community.

The life cycle of the *P. falciparum* parasite is characterized by three major stages: the pre-erythrocytic stage (divided into the sporozoite and liver phases), the blood (asexual) stage and the sexual stage. These three stages occur in two different hosts: humans and mosquitoes. Starting from a healthy individual (**Figure 2A**), the pre-erythrocytic stage of the infection is initiated by a bite from an infected female *Anopheles* sp. mosquito and the release of *P. falciparum* sporozoites into the skin and later into the blood of a human host (**Figure 2B**). The sporozoites are taken up by hepatocytes, resulting in the development of merozoites, which are released into the blood following hepatocyte rupture. The blood stage of the infection is characterized by a repetitive cycle of erythrocyte invasion, parasite replication and erythrocyte rupture, as well as the release of new merozoites. The clinical symptoms of malaria are stringently linked to this stage (**Figure 2C**). Only a small number of blood-stage parasites convert into the sexual form of the parasite (gametocytes) and can be taken up through a blood meal into a new mosquito host (**Figure 2D**). The sexual phase of the life cycle is initiated by the fusion of female and a male gametes resulting in the formation of oocysts. Sporozoite development in the oocysts perpetuates the life cycle and leads to multiple transmissions of the infection within a human community due to subsequent mosquito bites (**Figure 2E**; Ouattara and Laurens, 2015).

For the prevention of poverty-related diseases, millions of vaccine doses need to be produced at low cost (Waheed et al., 2012). Plant-based expression systems have the potential to match these requirements (Rybicki, 2010) and vaccine candidate antigens from several viral, bacterial and protozoan pathogens have been produced successfully using such platforms (Daniell et al., 2001; Clemente and Corigliano, 2012). The first plant-derived malaria vaccine candidate has recently commenced a phase I, dose escalation, first-in-human study (ClinicalTrials.gov Identifier: NCT02013687). Even so, the establishment of plant-based systems as a common production platform for vaccines is still hampered by issues with yields, DSP efficiency and regulatory compliance (Fischer et al., 2013).

In this DoE study we describe the optimization of DSP for a new pre-erythrocytic malaria vaccine candidate fusion protein (C9S) and a transmission-blocking vaccine candidate (FQS). Both proteins were produced by transient expression in *Nicotiana benthamiana* plants using the *Agrobacterium tumefaciens* vacuum infiltration method (Simmons et al., 2009). C9S is an optimized version of the CCT fusion protein, which has been developed and characterized as a heat-stable pre-erythrocytic malaria vaccine candidate (Voepel et al., 2014) and included as the pre-erythrocytic component of a plant-derived multi-component, multi-stage malaria vaccine cocktail (Boes et al., 2015). The “C” component of C9S comprises two TSR-domains from *Pf*CSP and *Pf*TRAP as well as the *Pf*Celtos antigen. This facilitates the induction of antibodies against the pre-erythrocytic stage of *P. falciparum*, potentially reducing the infection of liver cells and thereby the proliferation of the parasites within the liver. The components “9” and “Q” of C9S and FQS, respectively, are included to elicit blood-stage antigen-specific antibodies, preventing or reducing blood-stage parasitemia. The “F” component of FQS features a fusion protein of two transmission-blocking antigens from the sexual stage of the parasite, which are known to induce antibodies with strong transmission-blocking activity *in vitro* and in animal models (Beiss et al., 2015).

The FQS vaccine candidate is an optimized version of the F0 variant containing an additional epitope from a *P. falciparum* blood-stage antigen. The heat-stable fusion protein F0 has previously been included as the transmission-blocking component in the plant-derived multi-component, multi stage malaria vaccine cocktail mentioned above (Boes et al., 2015). Vaccines targeting each of the three stages in the parasite life cycle are promising candidates to increase overall protective efficacy by preventing or reducing the rate of primary infection (pre-erythrocytic stage), the manifestation of clinical symptoms (blood stage) and the rate of transmission (sexual stage).

## Materials and Methods

### Expression Constructs

DNA sequences encoding the fusion proteins C9S and FQS were obtained as synthetic genes (codon optimized for *N. benthamiana*) from Geneart (Thermo Fisher Scientific, Waltham, MA, USA). C9S comprises the pre-erythrocytic-stage antigens *Pf*CelTOS (PF3D7_1216600, F^25^-D^182^), *Pf*CSP_TSR (PF3D7_0304600, P^311^-S^383^), *Pf*TRAP_TSR (PF3D7_1335900, E^239^-K^289^; Voepel et al., 2014) and a part of the blood-stage antigen *Pf*RH5 (PF3D7_0424100, T^353^-H^365^; Douglas et al., 2014). FQS contains the sexual-stage antigens *Pfs*25 (PF3D7_1031000, V^24^-T^193^) and an N-terminal portion of *Pfs*230 (C0-fragment, PF3D7_0209000, E^443^-N^586^; Beiss et al., 2015), and a short region of the blood-stage antigen *Pf*RH5 (PF3D7_0424100, T^353^-H^365^; Douglas et al., 2014).

The synthetic genes were introduced into the binary plant expression vector pTRAkc-ER at the NcoI/NotI sites (Sack et al., 2015). The resulting ORFs encoded an N-terminal signal peptide sequence from the heavy chain of monoclonal antibody 24 (Voss et al., 1995) joined to the fusion protein C9S or FQS followed by a three-alanine linker and a C-terminal SEKDEL sequence to retrieve the protein to the ER. The final expression vectors were verified by sequencing and then introduced into *A. tumefaciens* by electroporation (Main et al., 1995).

### Plant Material and Infiltration

*Nicotiana benthamiana* plants were cultivated in a phytotron as previously described (Buyel et al., 2015a) at 25/22°C day/night temperature and 70% relative humidity. The plants were irrigated with 1.0 g L^-1^ Ferty 2 Mega (Planta Düngemittel GmbH, Regenstauf, Germany) for 15 min h^-1^ during the 16-h photoperiod (140 μmol s^-1^ m^-2^, λ = 400–700 nm) and were grown for 56 days prior to infiltration. The bacteria (*A. tumefaciens* strain GV3101:pMP90RK) were pre-cultured in sterile baﬄed Erlenmeyer flasks at 27°C shaking at 180 rpm for ∼48 h in YEB medium (5.0 g L^-1^ nutrient broth, 1.0 g L^-1^ yeast extract, 5.0 g L^-1^ peptone, 5.0 g L^-1^ sucrose, 0.5 g L^-1^ magnesium sulfate heptahydrate, pH 7.0) supplemented with 50 mg mL^-1^ carbenicillin, 25 mg mL^-1^ kanamycin, and 25 mg mL^-1^ rifampicin. We then inoculated 2 L of PAM medium (20 g L^-1^ soy peptone, 0.5 g L^-1^ yeast extract, 5.0 g L^-1^ fructose, 1.0 g L^-1^ magnesium sulfate heptahydrate, pH 7.0) supplemented with 50 mg mL^-1^ carbenicillin and 25 mg mL^-1^ kanamycin with the pre-cultures by adjusting the OD_600__nm_ to 0.1 and incubated the cultures for 65–72 h at 27°C in baﬄed flasks shaking at 160 rpm. The cultures were diluted with water and 2x infiltration medium (1.0 g L^-1^ Ferty 2 Mega) to an OD_600__nm_ of 1.0. The pH was adjusted to 5.6 with 12 M hydrochloric acid before infiltration. *N. benthamiana* plants without roots were then submerged in the suspension and subjected to a vacuum of 100 mbar for 1 min. The vacuum was released rapidly, non-infiltrated leaves were removed and the plants were drained briefly before transfer to an incubation chamber. After infiltration the plants were incubated under constant light (3× L58W/830 and 3× L58W/840; Osram Licht AG, Munich, Germany; 50 μmol s^-1^ m^-2^) for 5 days at ∼25°C and 70% relative humidity.

### Design of Experiments

Design Expert v9.0 (Stat-Ease, Minneapolis, MN, USA) was used to set up and evaluate the heat precipitation experiments in the extract and by blanching. Response surface models with 60 or 29 runs were used to characterize the heat precipitation experiments involving C9S and FQS, respectively. We investigated heating temperatures of 20–80°C, incubation times of 0–60 min, and a subsequent storage time at a RT of ∼20°C for up to 24 h in the case of C9S. Based on these results, the heat precipitation temperature was varied in the 65–80°C range for FQS. The amount of target protein per gram biomass was used as the response and normalized to the amount of target protein measured after heat precipitation at 80°C, with an incubation time of 5 min and a storage time of 0 h to compensate for batch-to-batch variations. A detailed description of the DoE approach is provided elsewhere (Buyel and Fischer, 2014a).

We characterized the blanching of intact leaves using I-optimal response surface designs with 52 and 26 runs for C9S or FQS, respectively. We investigated sodium phosphate concentrations of 0-40 mM in the blanching fluid, conductivities between 0.0162 (pure water) and 30.0 mS cm^-1^ adjusted with sodium chloride, the influence of the blanching time between 0.5 and 5 min and a storage time at RT of 0–24 h. The blanching was conducted at 80°C and the amount of target protein per gram biomass was normalized to the amount of target protein measured after 5 min blanching time, 40 mM sodium phosphate, a conductivity of 6.29 mS cm^-1^ resulting from the dissolved buffer and no storage at RT.

I-optimal response surface designs with 20 runs were used to study the pH stability of C9S and FQS. We adjusted the pH of heat-treated extract in the range 3.0–7.0 and monitored the amount of target protein per gram biomass over 24 h when stored at temperatures in the range 8–20°C.

### Heat Precipitation and Protein Extraction

#### Blanching

For blanching, a previously described procedure (Buyel et al., 2014) was used with modifications. Whole infiltrated *N. benthamiana* plants (∼100 g) without roots were plunged for up to 5 min into a pre-heated and agitated vessel containing 5 L of water or 40 mM sodium phosphate buffer, pH 8.0. The blanching temperature was set according to the DoE requirements. After blanching, the plants were drained for ∼15 s.

#### Protein Extraction

Proteins were extracted in 3 mL buffer (20 mM sodium phosphate, pH 8.0, 10 mM sodium bisulfite, conductivity adjusted to 15 mS cm^-1^ using sodium chloride) per gram of initial fresh mass, and mixed in a blender (Waring Products, East Windsor, NJ, USA) for 3 s × 30 s with interspersed 30 s residence periods. A sample from each extract (∼2 mL) was centrifuged (7 min, 16,000 × *g*, 4°C). After centrifugation, the supernatant was stored at RT for stability studies. Reduced samples for LDS-PAGE were prepared immediately after extraction and at different time points between 0 and 24 h.

#### Heat Treatment of Plant Extracts

An initial extraction was performed as described above. No sodium bisulfite was added for the extraction of FQS. Aliquots of 500 mL extract were transferred into a 0.78-kg, 2.0-L custom stainless-steel vessel (height 180 mm, diameter 120 mm). The vessel was placed in a pre-heated water bath (with a Lauda E300 heating circulator), and 2-mL samples of the homogenate were taken after reaching the temperatures and incubation times stated in the DoE. During heat precipitation, the homogenate was agitated and the temperature was controlled using thermometers (-10 to 150°C, VWR International, Radnor, PA, USA; -10 to 110°C, Carl Roth GmbH, Karlsruhe, Germany). The homogenate was centrifuged as above and stored for the different incubation times at RT. Samples for quantification of target protein and TSP were prepared as described above.

#### Heat Treatment during Extraction

Heated extraction in a blender was achieved by weighing plants and filling the glass vessel of the blender with three volumes of extraction buffer per gram biomass. The buffer and glass vessel (without biomass) were then heated in a water bath to ∼82°C. Then, the vessel was placed on the blender motor (Waring Products) and the plant material was added. Aluminum foil was used to insulate the glass vessel. Extraction, centrifugation, and sampling were carried out as described above.

### Measuring the pH Stability of C9S and FQS

Plant extracts were prepared as described above but in a buffer containing 20 mM sodium phosphate, 20 mM citric acid and 10 mM sodium bisulfite that was adjusted to pH 8.0 and a conductivity of 15.0 mS cm^-1^ using sodium chloride prior to extraction. No sodium bisulfite was added to the extraction buffer for FQS. After extraction, heat precipitation was carried out in a steel vessel as described above and the extract was centrifuged (20 min, 38,400 × *g*, 4°C). The pH was then adjusted as required for the DoE using 10 M sodium hydroxide or 12 M hydrochloric acid. Aliquots of the homogenate were stored at 8, 14, and 20°C and samples were taken at the time points defined by the DoE. Samples for LDS-PAGE were centrifuged (7 min, 16,000 × *g*, 4°C) and immediately heated to 70°C in LDS-PAGE loading buffer (Thermo Fischer Scientific). Samples for TSP analysis were stored at -80°C.

### Identification of Protease Activity

Proteins were extracted from infiltrated *N. benthamiana* plants as described above and protease inhibitors were added directly to the extract at 20°C or after heat treatment at 50, 60, or 80°C. We used final concentrations of 10 μM E-64 (*trans*-epoxysuccinyl-L-leucylamido(4-guanidino)butane), 1 mM phenylmethylsulfonylfluoride (PMSF), 5 μM pepstatin and 5 mM 1,10 Phen. Alternatively, one tablet of cOmplete protease inhibitor mix (Roche, Basel, Switzerland) was directly dissolved in 25 mL of plant extract. Product degradation was monitored at RT for 24 h by immunoblot analysis (see below).

### Protein Quantitation

The quantity of TSP in the supernatants was determined by the Bradford method (Simonian and Smith, 2006) using 2.5 or 5.0 μL aliquots as previously described (Buyel and Fischer, 2014c). Eight BSA dilutions in the range 0–2 mg mL^-1^ were used to build a standard curve to calculate the TSP concentrations. The target proteins were quantified in 4–12% Bis-Tris polyacrylamide gels (Thermo Fischer Scientific) stained with SimplyBlue Safe Stain (Thermo Fischer Scientific) and subsequent densitometric analysis using the AIDA image analyzer software (Raytest, Straubenhardt, Germany). Solutions of His_6_-tagged product variants were used as references. These standard proteins were purified by immobilized metal affinity chromatography (IMAC) as previously described (Voepel et al., 2014) but with copper ions as the ligand instead of nickel.

### Product Characterization

The molecular mass and isoelectric point of C9S and FQS were calculated from the amino acid sequence using Clone Manager (Scientific & Educational Software, Morrisville, NC, USA).

C9S was detected using 0.4 μg mL^-1^ of a *Pf*TRAP_TSR domain-specific monoclonal antibody (Fraunhofer IME, Aachen, Germany). FQS was detected using the reduction-sensitive monoclonal antibody 4B7 (Malaria Research and Reference Reagent and Resource Center, Manassas, VA, USA) diluted 1:5000 in phosphate buffered saline (PBS). A polyclonal goat anti-mouse IgG labeled with alkaline phosphatase (Jackson ImmunoResearch, West Grove, PA, USA) was used as the secondary antibody, diluted 1:5000 in PBS. The signal was detected with 0.3 mg mL^-1^ nitro blue tetrazolium (NBT) and 0.16 mg mL^-1^ 5-bromo-4-chloro-3-indolyl phosphate (BCIP) in alkaline phosphatase buffer (100 mM Tris-Cl, 100 mM sodium chloride, 5 mM magnesium chloride, pH 9.6). The target band intensity was quantified using AIDA image analyzer software and the corresponding His_6_-tagged product as a reference (42–250 ng per lane).

## Results

### The Properties of Malaria Vaccine Candidates C9S and FQS and their Expression in *N. benthamiana*

**FIGURE 3:**
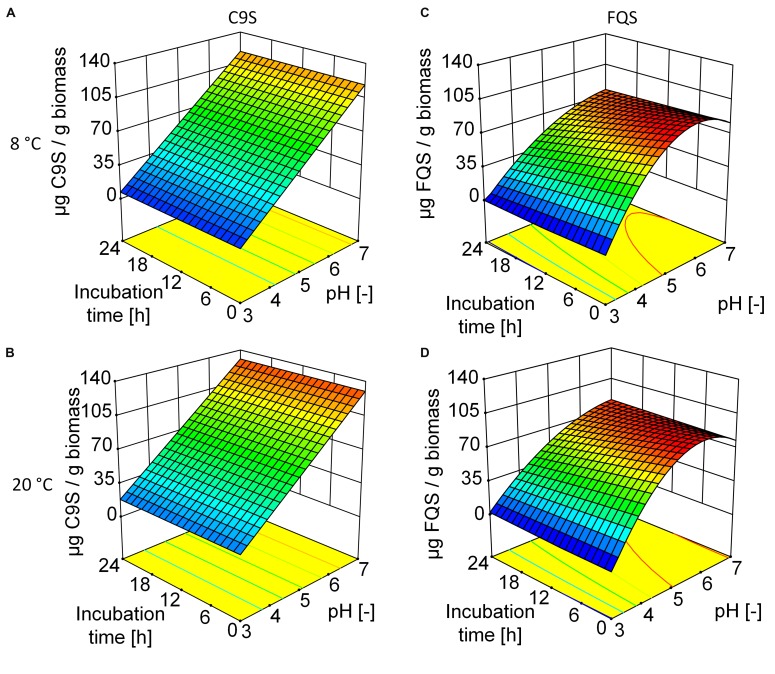
**Response surfaces models illustrating the effect of pH, storage time and temperature on the yield of C9S (A,B) and FQS (C,D).** The yield of both target proteins decreased at lower pH values whereas incubation time and temperature had no relevant effect. At pH 5.0, the yield of FQS was higher than that of C9S. Colors are used to indicate high (red) or low (blue) response values.

The C9S and FQS vaccine candidates were expressed in *N. benthamiana* plants resulting in average expression levels of 92.2 ± 6.1 (*n* = 11) mg kg^-1^ and 48.9 ± 7.8 (*n* = 2) mg kg^-1^, respectively. We predicted the sizes and isoelectric points of both proteins based on their amino acid sequences (C9S = 35 kDa and pI 4.7, FQS = 37 kDa and pI 4.3). As anticipated based on these pI values, the solubility of both proteins decreased as the pH was reduced to ∼4.0, ultimately resulting in the loss of 85% C9S and 100% FQS at pH 3.0 (**Figure 3**) and causing more of each protein to appear in the pellet fraction after centrifugation (data not shown). The DoE evaluation revealed a linear correlation between pH and the solubility of C9S and a quadratic response was observed for FQS (Supplementary Tables S1 and S2). Incubation temperature and duration did not have a relevant effect on the solubility of either target protein.

**FIGURE 4:**
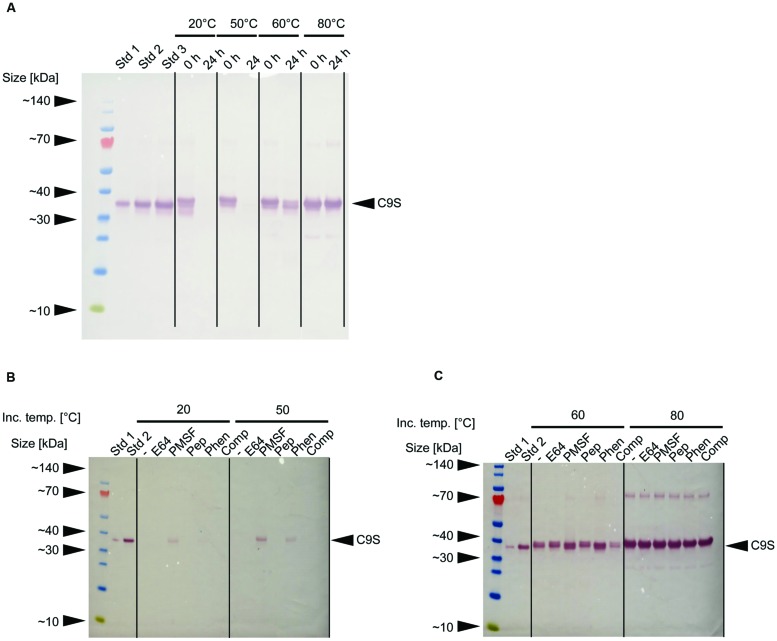
**Effect of heat treatment and protease inhibitors on the yield of malaria vaccine candidate C9S. (A)** Immunoblot of C9S using a mouse-derived monoclonal primary antibody against *Pf*TRAP_TSR and alkaline phosphatase labeled goat-anti-mouse secondary antibody immediately after the heat treatment of plant extracts at different temperatures and after storage at RT for 24 h. **(B,C)** Immunoblots using the same antibodies as above, showing the effect of protease inhibitors on the yield of C9S after the plant extracts were treated at different temperatures and stored at RT for 24 h. Std 1–3 denote His_6_-tagged protein variants used for quantification: 83, 166, and 250 ng per lane **(A)** or 42 and 166 ng per lane **(B,C)**.

In our initial experiments, neither target protein was detected in samples of clarified supernatant after storage at RT for 24 h (data not shown). Immunoblot analysis revealed that the loss of C9S could be avoided if the extracts were heated to 60–80°C for 5 min before storage at RT for 24 h (**Figure 4A**). Furthermore, a combination of heat treatment at 50°C and the inclusion of certain protease inhibitors partially restored the yield of C9S to ∼9% (Phen) and ∼22% (PMSF) of the level observed after heat treatment at 80°C. Only PMSF preserved detectable amounts of C9S after heat treatment at 20°C and storage at RT for 24 h, achieving a yield of ∼10% compared to the level observed after heat treatment at 80°C (**Figures 4B,C**). We did not conduct the corresponding analysis of FQS.

### Improving the Stability of C9S and FQS at Neutral pH by Heat Precipitation

**FIGURE 5:**
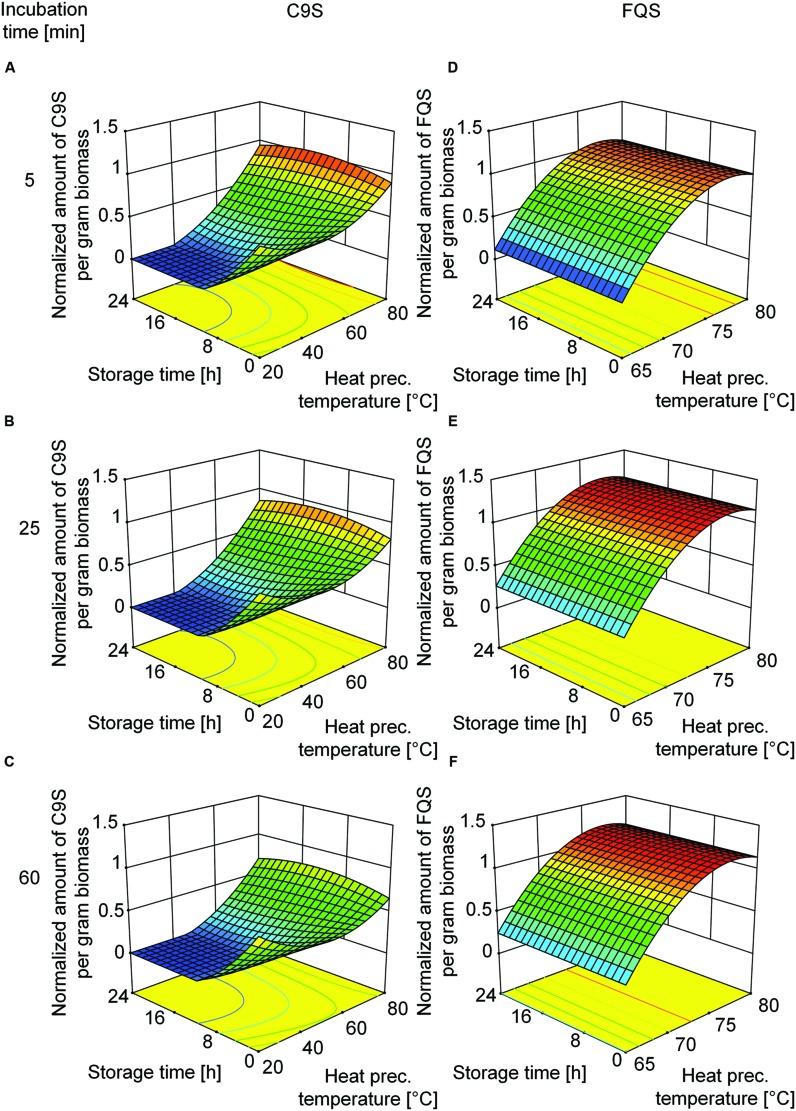
**Response surface representations of predictive models for C9S and FQS yields after the heat treatment of plant extracts, showing dependence on incubation temperature as well as incubation and storage durations.** The yields of C9S **(A–C)** decreased if the heat treatment temperature fell below 65°C and losses increased with longer incubation and storage times. The yields of FQS **(D–F)** decreased if the heat treatment temperature fell below 72°C but neither incubation nor storage time had a relevant effect. C9S and FQS yields were normalized to a reference condition based on blanching at 80°C for 5 min and subsequent storage for 0 h. Colors are used to indicate high (red) or low (blue) response values.

Based on the beneficial effect of heat treatment on target protein yield, we decided to investigate the underlying mechanism in more detail using a DoE approach. We used the heat precipitation temperature and the incubation time during heat precipitation as the major factors. Analysis of the corresponding DoE profiles (Supplementary Tables S2 and S3) revealed that the highest yields of C9S and FQS were achieved at incubation temperatures in the 75–80°C range (**Figure 5**). The yield of FQS was not affected by prolonged incubation times at these temperatures, but the yield of C9S declined by ∼33% if the incubation time was increased from 5 to 60 min. In contrast, both products were completely lost after 24 h if the heat treatment step was carried out at less than 50°C (C9S) or 65°C (FQS; **Figure 5**).

### Investigation of Different Heat Precipitation Methods

**FIGURE 6:**
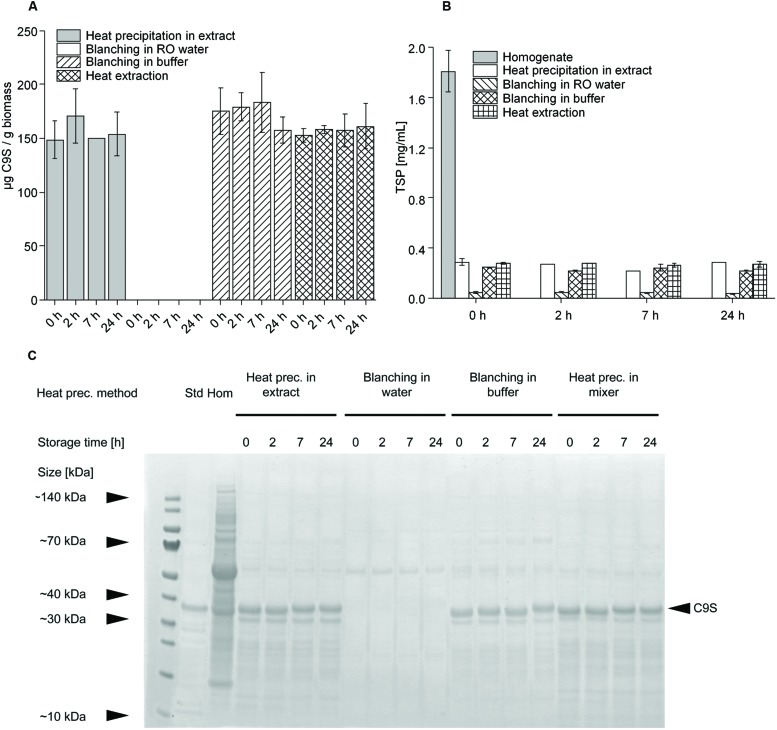
**Comparison of the influence of different heat precipitation methods (heat precipitation, blanching in water, blanching in buffer, heat extraction) at 80°C on target protein concentrations (A,C) and TSP levels (B).** Target protein stability was achieved by heat precipitation in extract, blanching in buffer and heat extraction. The band representing target protein C9S in the Coomassie-stained gel shown in panel **(C)** is indicated with a black arrow.

Having established that heat treatment for 5 min at 80°C achieved the highest yields of C9S and FQS we set out to investigate other heat precipitation methods that might be easier to integrate into the production process. Therefore, we compared heat precipitation applied to plant extracts with the blanching of intact plants (Buyel et al., 2014) using water or buffer, and with heat precipitation using hot buffer during protein extraction. C9S was selected as the model protein for this experiment. We observed no significant difference (two-sided *t*-test, α level = 0.05) in the yield of C9S when we compared heat precipitation in plant extract, blanching in buffer and extraction in hot buffer (**Figure 6A**). There was also no significant loss of product in the subsequent 24-h incubation period at RT when using any of the three heating methods, achieving average yields of 153.9 ± 19.2 mg kg^-1^ (*n* = 10). In contrast, no C9S was detected in the plant extracts at any time if blanching was carried out in water instead of buffer. Blanching in water reduced the amount of TSP by ∼97%, resulting in a final concentration of ∼0.05 mg mL^-1^ (**Figure 6B**). The other three methods reduced the TSP by ∼85%, again with no significant difference among them and without the TSP concentration changing over the 24-h incubation period. Analysis by LDS-PAGE revealed that the lower amount of TSP in the heat-treated samples was clearly linked to the removal of HCPs (**Figure 6C**), including ribulose-1,5-bisphosphate carboxylase/oxygenase (RuBisCO). The removal of HCPs increased the purity of C9S from 0.02 g g^-1^ TSP in the untreated homogenate to 0.15 g g^-1^ TSP following the heat treatment of extract, blanching in buffer or extraction in hot buffer.

### Influence of Blanching Time and Buffer Composition on C9S and FQS Yields

**FIGURE 7:**
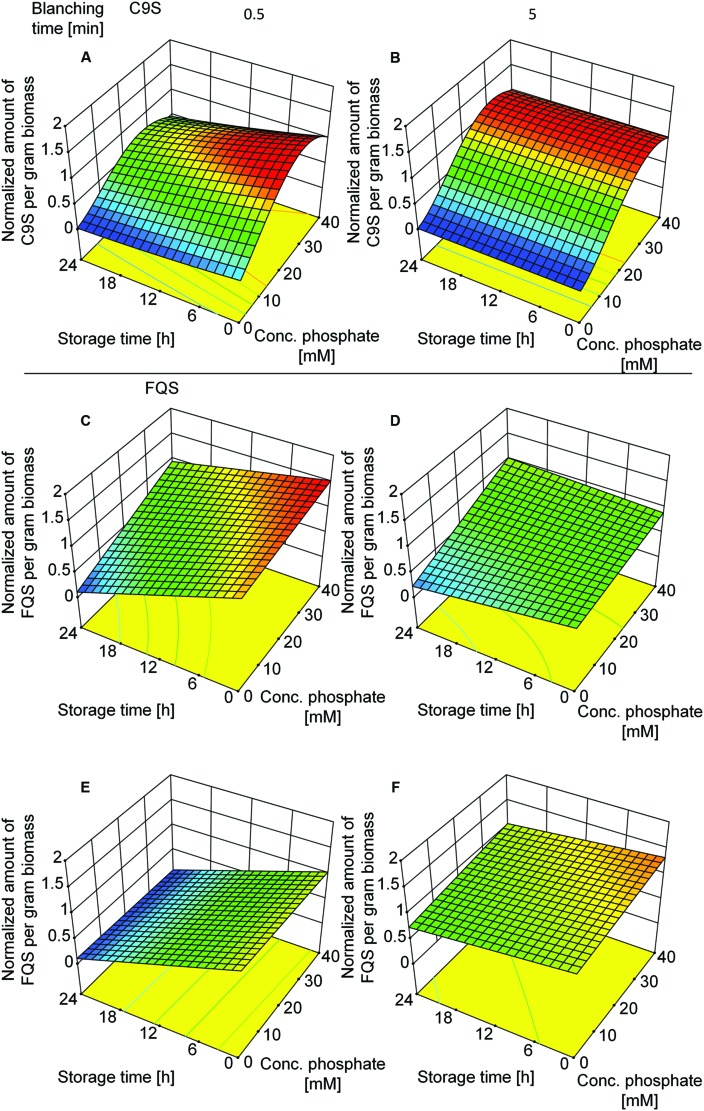
**Response surface models showing how the yields of C9S (A,B) and FQS (C–F) depend on phosphate concentration in the blanching buffer, incubation and storage times as well as buffer conductivity.** A phosphate concentration of 30–40 mM resulted in the highest yields of C9S and an incubation time of more than 0.5 min was required for the C9S to concentration to remain constant over a 24 h incubation period at RT. The phosphate concentration did not have a relevant effect on the yield of FQS but incubation times exceeding 0.5 min were necessary to prevent product loss over the subsequent 24 h incubation period. Colors are used to indicate high (red) or low (blue) response values.

Given that C9S could be extracted from plants blanched in buffer but not from those blanched in water, we decided to investigate the effect of buffer composition, i.e., phosphate concentration and conductivity. We therefore set up a DoE approach including both vaccine candidates and tested the effect of buffer composition and the duration of blanching. The buffer concentration was the dominant factor in the case of C9S (Supplementary Table S5) and the highest yields of 156.4 ± 21.6 (*n* = 13) μg g^-1^ biomass were achieved at phosphate concentrations in the range 30–40 mM. In contrast, the phosphate concentration had a smaller impact on the yield of FQS (Supplementary Table S6). The duration of blanching only became important when the blanching step was brief, i.e., both C9S and FQS were completely lost during post-extraction storage at RT for 24 h only if the duration of blanching was reduced to 0.5 min (**Figure 7**). Increasing the time to 5.0 min restored the yield to 156.4 ± 21.6 (*n* = 13) μg g^-1^ for C9S and 50 ± 13.8 (*n* = 5) μg g^-1^ for FQS.

### Comparison of Heat Precipitation and Blanching

**FIGURE 8:**
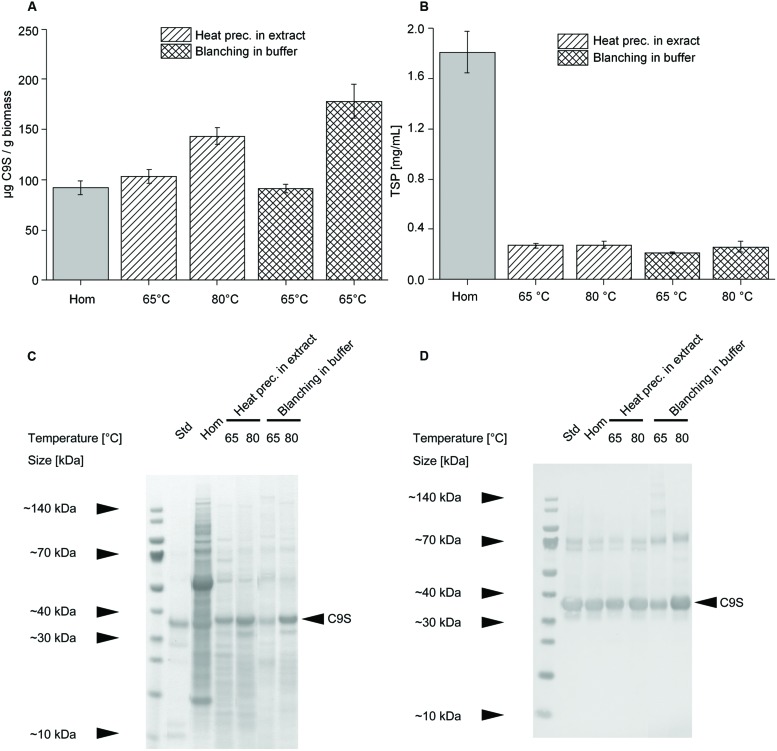
**Comparison of blanching in buffer and heat precipitation in the plant extract.** The yield of C9S increased for both methods when heat treatment was carried out at 80°C instead of 65°C **(A)**, whereas there was no difference in TSP levels at these temperatures, i.e., both methods achieved a reduction of 85% compared to untreated extract **(B)**. The increased yields were also observable on Coomassie-stained LDS-PAGE gels **(C)** and western blots using C9S-specific primary antibody for detection **(D)**. Hom – homogenate; Std – His_6_-tagged C9S variant, 390 ng per lane.

As stated above, heat treatment inhibited target protein degradation and reduced the TSP content by more than 85% (**Figure 8B**). Heat precipitation in a vessel at 65°C achieved C9S yields of 103.5 ± 7 (*n* = 3) μg g^-1^ biomass, increasing by 40% to 143.6 ± 8.4 (*n* = 3) μg g^-1^ biomass at 80°C (**Figure 8A**). Similarly, blanching at 65°C achieved C9S yields of 91.4 ± 8.4 (*n* = 3) μg g^-1^ biomass, increasing by 100% to 178.1 ± 17 (*n* = 3) μg g^-1^ biomass at 80°C (**Figure 8A**). Blanching therefore appeared to be more efficient for the recovery of C9S from the plant biomass. In contrast, both methods removed tobacco HCPs with similar efficiency of >85% at both temperatures, as shown by monitoring the TSP content (**Figure 8B**). Immunoblot analysis using an antibody specific for the *Pf*TRAP_TSR domain of C9S revealed a dominant band with an apparent molecular mass of ∼35 kDa (**Figure 8D**) which was also the dominant band on corresponding gels stained with Coomassie Brilliant Blue (**Figure 8C**).

## Discussion

### The Properties of Each Target Product Influence DSP Development

Many tobacco HCPs have pI values greater than 6.5 (Buyel and Fischer, 2014d). Therefore, cation exchange (CEX) chromatography can be a useful HCP-removal step if the target protein has a pI below 6.0 and no affinity resin is available, as was the case for our malaria vaccine candidates C9S and FQS. CEX chromatography typically requires a buffer pH below 6.0 to work effectively (Ge Healthcare, 2010). This pH can also be used to precipitate several tobacco HCPs, including RuBisCO (Hassan et al., 2008; Buyel and Fischer, 2014b), thereby increasing the purity of the product before the first chromatography step, which can facilitate subsequent unit operations (Winkelnkemper and Schembecker, 2010). We therefore tested the two target proteins to determine their stability at pH ∼7.0, which is typically used for extraction (Hassan et al., 2008), and also in the pH range 3.0–5.0. Unfortunately, both proteins precipitated at pH values below 5.0, reducing the yields by 50% or more. We therefore concluded that low-pH conditions suitable for the precipitation of HCPs and typical for CEX loading buffers were not compatible with C9S or FQS. Furthermore, both proteins were unstable during storage and their concentrations fell below our detection threshold of ∼1 μg mL^-1^ after incubation at RT for 24 h. Indeed, losses exceeding 50% were observed after ∼7 h, a typical duration of a hold step prior to product capture in large-scale production processes.

We anticipated that host cell proteases are likely to be responsible for the observed losses so we supplemented the plant extracts individually with E-64 to inhibit cysteine proteases, PMSF to inhibit serine proteases, pepstatin to inhibit aspartyl proteases and Phen to inhibit metalloproteases. PMSF, and to a lesser extent Phen, partially inhibited the degradation of C9S indicating that this protein is susceptible to the combined action of serine proteases and metalloproteases, which are known to be present in plant extracts as active enzymes (Mandal et al., 2014).

Our previous work with malaria vaccine candidates has shown that thermal stability can be an inherent feature of *P. falciparum* surface antigens and fusions thereof, and can be exploited for the heat-induced selective removal of tobacco HCPs in the temperature range 60–80°C (Buyel et al., 2014; Voepel et al., 2014; Beiss et al., 2015). We therefore investigated whether the heat treatment of extracts containing C9S or FQS could remove the HCPs and inactivate the proteases at the same time, thereby increasing the recovery and stability of the target proteins.

### Heat Precipitation of Tobacco HCPs Increases Product Stability

Based on our earlier work, we tested the efficiency of HCP precipitation by heating the tobacco extracts to 20–80°C for 5–60 min. The concentrations of C9S and FQS in extracts heated to 75–80°C remained constant when the samples were maintained at RT for the subsequent 24 h. However, the recovery of C9S became less efficient if the heat treatment was prolonged beyond 5 min, indicating that C9S is less stable than FQS. This indicates that heating should be kept to a minimum duration even for proteins designed to be heat stable, because losses can occur over time as reported for another malaria vaccine candidate, Vax8 (Buyel et al., 2014).

When we compared different heat treatment methods, we found that they performed equally well in terms of removing tobacco HCPs and preventing product losses due to protease activity. However, when the blanching buffer (pH 8.0) was replaced with water, we observed the complete loss of C9S (but not FQS). We propose that this phenomenon ultimately reflects the rupture of plant cell membranes, especially the vacuolar membrane, by the osmotic shock caused by heating in water, resulting in the homogenization of the formerly separate intracellular compartments. The vacuole represents the largest proportion of the cytosol by volume, accounting for ∼90% of the total (Owens and Poole, 1979). Because the vacuole is acidic, the pH of the resulting cell lysate falls to ∼5.4 (Thoday and Evans, 1932; Buyel and Fischer, 2014e), which as discussed above causes the precipitation of C9S along with many tobacco HCPs but leaves ∼50% of FQS in solution. The use of a buffer during blanching prevents the pH drop caused by cell lysis and thus avoids the precipitation of proteins such as C9S and several HCPs that are sensitive to low pH values. This hypothesis is supported by our observation that the pH of the blanching buffer becomes lower as more plants are blanched, requiring periodic readjustment to pH 8.0 (data not shown). The proposed mechanism also explains why the yield of C9S was dependent on the concentration of phosphate in the blanching buffer: the higher the concentration of phosphate, the higher the buffering capacity and thus the smaller the pH drop induced by membrane rupture.

The membrane of the ER may also be affected by rupture suggesting that the product could diffuse from the plant tissue and be lost in the blanching fluid, reducing the process yield. We found no significant difference in C9S yields achieved with blanching in buffer, extraction in hot buffer or heat precipitation after extraction (**Figure 4A**). This indicated that if product losses occurred due to the rupture of the ER membrane, they were negligible compared to the experimental error of ±10% (m/m; *n* = 12) we observed for the C9S concentration. We speculate that the product yield was not affected by blanching-induced membrane rupture because the plant tissue, including the cell wall and remaining membrane fragments, was a diffusion barrier for the proteins but not for the hydronium ions (H_3_O^++^) responsible for the observable change in blanching buffer pH. The hydrodynamic radius of hydronium ions is 0.143 nm (Choi et al., 2005), which is smaller by a factor of 15 than the radius of a 35 kDa protein, such as C9S, which is ∼2.16 nm (Erickson, 2009).

### Process Considerations for the Choice of Heat Precipitation Method

The application of statistical experiment planning using dedicated DoE software and thorough data analysis allowed the rapid characterization of the different heat precipitation methods and target protein pH stability. The resulting descriptive models were of high quality, i.e., *R^2^* > 0.9 (**Table 1**), and were used for process optimization.

**Table 1:** Quality parameters of the predictive models for heat precipitation in extract, blanching in buffer and the pH stability of C9S and FQS derived from DoE analysis.

Model	Target protein	*R*^2^	Adjusted *R*^2^	Predicted *R*^2^
Heat precipitation in extract	C9S	0.9051	0.8903	0.8441
	FQS	0.9806	0.9773	0.9688
Blanching in buffer	C9S	0.9736	0.9686	0.9548
	FQS	0.9763	0.9545	0.8132
pH stability	C9S	0.9664	0.9624	0.9537
	FQS	0.9551	0.9923	0.9836

The product yield is an important aspect to consider when selecting a heat precipitation method. Here, the highest yields were achieved by blanching intact plants in a buffer. Another key issue is the ease with which a specific operation can be integrated into a DSP, particularly an existing process. The heating of plant extracts initially seems an appealing option given that stirred vessels with integral heating jackets are readily available in many laboratories. However, this approach has several drawbacks including the large volumes of buffer that must be heated and cooled (and the corresponding high energy costs) and the declining surface-to-volume ratio during scale-up, which reduces the relative heat transfer (Buyel et al., 2014). These issues do not affect blanching because this process requires a single step to heat a defined volume of buffer, and only a small amount of additional energy is required to heat the plant biomass immersed in the fluid. The use of hot buffer during extraction reduces the process time by integrating extraction and heat precipitation. However, it can take several minutes to add hot buffer to a blender in a scaled-up process, so some of the biomass may be in contact with the hot buffer for longer than anticipated, risking the loss of sensitive products such as C9S. Cooling also becomes more challenging at larger scales because the surface-to-volume ratio becomes progressively smaller. In any case, knowledge of the thermal properties of the plant biomass, i.e., specific heat capacity and thermal conductivity is important for the design of the equipment used for heating (Buyel et al., 2016). This design process can be facilitated by numeric modeling approaches that can provide valuable insights into the heat transfer mechanisms as has been recently described for blanching (Buyel, 2016).

Overall, blanching appeared to be the most suitable technique for plant HCP removal. The method increased the concentration of C9S from 2 to 15% (m/m) TSP. Hence, fewer HCPs would be available to bind during an initial capture step, most likely using an ion exchange resin. This would save binding capacity for the actual target protein, potentially reducing the column size by a factor of 7.5 and investment costs would decline accordingly. Furthermore, the lower number of HCP species binding during capture would facilitate separation of the HCPs from the product because co-elution is less likely. As a consequence, a smaller number of purification steps would be sufficient to achieve the required final purity. On the other hand, heat treatment potentially has a negative impact on the capacity of depth filters used during clarification (Buyel et al., 2014), but countermeasures such as the use of flocculants can be implemented to restore filter performance (Buyel and Fischer, 2014c).

## Conclusion

We have demonstrated how the rigorous analysis and characterization of heat precipitation methods cannot only remove the majority of HCPs from crude plant extract and thus represent a relevant simplification of subsequent DSP steps, especially for target proteins that cannot be captured using affinity-based methods, but also how heat treatment can increase the recovery of product from biomass and the stability of target proteins after extraction by inactivating host cell proteases present in the crude extract. A prerequisite for the successful application of these methods is the heat stability of the target protein. Further investigation will be necessary to confirm the functionality of the purified vaccine candidates C9S and FQS with respect to the correct presentation of relevant epitopes in animal immunization studies and subsequent *in vitro* parasite inhibition assays.

## Author Contributions

SM setup and conducted the blanching and heat precipitation experiments along with sample and DoE analysis. TH was involved in DoE planning and evaluation. AB contributed the initial systems setup and cloning. HS was responsible for gene cloning and writing the manuscript. JB setup and conducted pH-stability experiments and extraction in hot buffer. RF was involved in data analysis and interpretation. JFB was responsible for DoE evaluation, data interpretation and manuscript writing.

## Conflict of Interest Statement

The authors declare that the research was conducted in the absence of any commercial or financial relationships that could be construed as a potential conflict of interest. The authors declare that AB, HS, and RF have filed a patent application related to the recombinant vaccine candidate proteins C9S and FQS entitled “Multi-component-multi-stage malaria vaccine” (application number: 140011550.2–1412).

## Abbreviations

BSA: bovine serum albumin
Comp: cOmplete protease inhibitor cocktail
DoE: design of experiments
DSP: downstream processing
ER: endoplasmic reticulum
GMP: good manufacturing practice
HCP: host cell protein
OD_600 nm_: optical density at a wavelength of 600 nm
Phen: 1,10 phenanthroline
QbD: quality by design
rpm: revolutions per minute
RT: room temperature
TSP: total soluble protein
USP: upstream production
YEB: yeast extract broth
