# Supplementary material for: Optimized Blanching Reduces the Host Cell Protein Content and Substantially Enhances the Recovery and Stability of Two Plant-Derived Malaria Vaccine Candidates
Source: Front Plant Sci. 2016 Feb 17;7:159. doi: 10.3389/fpls.2016.00159 (PMC4756251; doi:10.3389/fpls.2016.00159)
Supplement: Supplementary file 1 [file Table_1.PDF]

Supplementary materials

Table S1: Model factors with significant impact on the pH-stability of C9S.

Source	Sum of squares	Degrees of freedom	F-value	P-value
Model	43769.60	2	244.15	<0.0001
pH [-] (A)	42783.41	1	477.31	<0.0001
Storage temperature [°C] (B)	414.42	1	4.62	0.0462
Residual	1523.80	17	n.a.	n.a.
Lack of fit	1373.05	12	3.80	0.0755
Pure error	150.75	5	n.a.	n.a.

Table S2: Model factors with significant impact on the pH-stability of FQS.

Source	Sum of squares	Degrees of freedom	F-value	P-value
Model	25796.30	7	305.05	<0.0001
pH [-] (A)	20535.78	1	1950.65	<0.0001
Storage time [h] (B)	446.03	1	42.37	<0.0001
Storage temperature [°C] (C)	0.77	1	0.073	0.7913
AB	0.0034	1	0.0003229	0.9860
BC	34.42	1	3.27	0.0957
A^2	2901.97	1	275.65	<0.0001
A^2B	333.52	1	31.68	0.0001
Residual	126.33	12	n.a.	n.a.
Lack of fit	37.16	7	0.30	0.9273
Pure error	89.17	5	n.a.	n.a.

Table S3: Model factors significantly affecting the recovery of C9S after heating the plant extract based on DoE analysis of variance.

Source	Sum of squares	Degrees of freedom	F-value	P-value
Model	5.53	8	60.83	<0.0001
Heat precipitation temperature [°C] (A)	0.52	1	45.70	<0.0001
Incubation time [min] (B)	0.044	1	3.91	<0.0533
Storage time [h] (C)	0.85	1	74.97	<0.0001
AB	0.058	1	5.07	0.0287
AC	0.75	1	65.66	<0.0001
A^2	0.55	1	48.63	<0.0001
C^2	0.055	1	4.81	0.0329
AC^2	0.14	1	12.25	0.0010
Residual	0.58	51	n.a.	n.a.
Lack of fit	0.14	24	2.68	0.0073
Pure error	0.17	27	n.a	n.a.

Table S4: Model factors significantly affecting the recovery of FQS after heating the plant extract based on DoE analysis of variance.

Source	Sum of squares	Degrees of freedom	F-value	P-value
Model	2.99	4	302.72	<0.0001
Temperature during heating [°C] (A)	2.85	1	1152.74	<0.0001
Incubation time during heating [min] (B)	0.13	1	54.60	<0.0001
A^2	0.55	1	23.92	<0.0001
B^2	0.059	1	14.51	0.00190
Residual	0.059	24	n.a.	n.a.
Lack of fit	0.011	12	4.56	0.0068
Pure error	41.06	12	n.a.	n.a.

Table S5: Model factors significantly affecting the recovery of C9S after blanching intact plants and subsequent extraction based on DoE analysis of variance.

Source	Sum of squares	Degrees of freedom	F-value	P-value
Model	6.83	8	193.91	<0.0001
Phosphate concentration [mM] (A)	3.30	1	749.20	<0.0001
Conductivity [mS cm^{-1}] (B)	0.18	1	40.93	<0.0001
Blanching time [min] (C)	0.00218	1	0.50	0.4854
Storage time [h] (D)	0.22	1	49.32	<0.0001
AB	0.32	1	72.51	<0.0001
AC	0.29	1	65.04	<0.0001
CD	0.20	1	44.78	<0.0001
A^2	0.88	1	201.6	<0.0001
Residual	0.18	42	n.a.	n.a.
Lack of fit	0.13	26	1.49	0.2064
Pure error	0.054	16	n.a.	n.a.

Table S6: Model factors significantly affecting the recovery of FQS after blanching intact plants and subsequent extraction based on DoE analysis of variance.

Source	Sum of squares	Degrees of freedom	F-value	P-value
Model	4.91	12	44.70	<0.0001
Phosphate concentration [mM] (A)	0.83	1	90.24	<0.0001
Conductivity [mS cm^{-1}] (B)	0.001279	1	0.14	0.7126
Blanching time [min] (C)	0.0005856	1	0.064	0.8042
Storage time [h] (D)	1.66	1	181.43	<0.0001
AB	0.32	1	35.35	<0.0001
AC	0.011	1	1.25	0.2835
AD	0.18	1	19.68	0.0007
BC	0.86	1	94.21	<0.0001
BD	0.039	1	4.25	0.0599
CD	0.67	1	72.83	<0.0001
ABC	0.064	1	6.96	0.0205
ABD	0.12	1	12.74	0.0034
Residual	0.12	13	n.a.	n.a.
Lack of fit	0.043	3	1.90	0.1942
Pure error	0.076	10	n.a.	n.a.
